# Endosulfine alpha maintains spindle pole integrity by recruiting Aurora A during mitosis

**DOI:** 10.1186/s12885-023-11742-0

**Published:** 2023-12-21

**Authors:** Seul Kim, Kyoungho Jun, Ye-Hyun Kim, Kwan-Young Jung, Jeong Su Oh, Jae-Sung Kim

**Affiliations:** 1 Division of Radiation Biomedical Research, Korea Institute of Radiological and Medical Sciences, 215-4 Gongneung-Dong, Nowon-Ku, Seoul, 139706 Korea; 2 Radiological and Medico-Oncological Sciences, University of Science and Technology, Daejeon, 34113 Korea; 3 Therapeutics & Biotechnology Division, Korea Research Institute of Chemical Technology, Daejeon, 34114 Korea; 4 Department of Integrative Biotechnology, Sungkyunkwan University, Suwon, 16419 Korea

**Keywords:** ENSA, Mitosis, Spindle pole integrity, MASTL, Aurora A

## Abstract

**Background:**

The maintenance of spindle pole integrity is essential for spindle assembly and chromosome segregation during mitosis. However, the underlying mechanisms governing spindle pole integrity remain unclear.

**Methods:**

ENSA was inhibited by siRNA or MKI-2 treatment and its effect on cell cycle progression, chromosome alignment and microtubule alignment was observed by immunohistochemical staining and western blotting. PP2A-B55α knockdown by siRNA was performed to rescue the phenotype caused by ENSA inhibition. The interaction between ENSA and Aurora A was detected by in situ PLA. Furthermore, orthotopic implantation of 4Tl-luc cancer cells was conducted to confirm the consistency between the in vitro and in vivo relationship of the ENSA-Aurora A interaction.

**Results:**

During mitosis, p-ENSA is localized at the spindle poles, and the inhibition of ENSA results in mitotic defects, such as misaligned chromosomes, multipolar spindles, asymmetric bipolar spindles, and centrosome defects, with a delay in mitotic progression. Although the mitotic delay caused by ENSA inhibition was rescued by PP2A-B55α depletion, spindle pole defects persisted. Notably, we observed a interaction between ENSA and Aurora A during mitosis, and inhibition of ENSA reduced Aurora A expression at the mitotic spindle poles. Injecting MKI-2-sensitized tumors led to increased chromosomal instability and downregulation of the MASTL-ENSA-Aurora A pathway in an orthotopic breast cancer mouse model.

**Conclusions:**

These findings provide novel insights into the regulation of spindle pole integrity by the MASTL-ENSA-Aurora A pathway during mitosis, highlighting the significance of ENSA in recruiting Aurora A to the spindle pole, independent of PP2A-B55α.

**Supplementary Information:**

The online version contains supplementary material available at 10.1186/s12885-023-11742-0.

## Introduction

Centrosomes are primary microtubule-organizing centers within cells and are important for the formation and function of spindle poles [1, 2]. Spindle poles orchestrate the organization of microtubules and facilitate the precise movement of chromosomes during cell division [1, 2]. Maintaining the integrity of the spindle pole is crucial for the correct segregation of chromosomes during cell division, as defects can lead to genetic abnormalities or cell death. Such disturbances contribute to chromosomal instability (CIN), a characteristic often associated with the development and progression of cancer [3].

Aurora A is a protein kinase that localizes to the spindle poles during mitosis and plays a key role in the proper formation of the mitotic spindle [4]. This is accomplished by the recruitment of crucial proteins including TACC3, TPX2, and NuMA, which are essential for establishing functional spindle structure [5–7]. Aurora A is also indispensable for the separation of the centrosome prior to spindle assembly and the organization and alignment of chromosomes during mitotic progression [8, 9]. Therefore, Aurora A at the spindle poles is in the prime position to coordinate mitotic events, and understanding the regulatory mechanisms underlying the recruitment of Aurora A to the spindle poles during mitosis is important for understanding genetic abnormalities such as CIN in cancer.

Endosulfine alpha (ENSA) is a major substrate protein for mitotic kinase microtubule-associated serine/threonine-protein kinase-like (MASTL) [10, 11]. MASTL plays a crucial role in regulating PP2A-B55 phosphatase during mitosis [12–15]. Phosphorylation of ENSA (p-ENSA) by MASTL inactivates PP2A-B55 at the onset of mitosis, facilitating maximal cyclin-dependent kinase (CDK) activity during mitosis [12–14]. This finding emphasizes the essential role of the MASTL-ENSA-PP2A-B55 pathway in the control of mitotic entry and exit. However, mitotic defects in MASTL-depleted cells can be partially rescued by depleting PP2A-B55 in human cancer cells [13, 16], implying that PP2A-B55 is not the only substrate of the MASTL pathway. Another substrate of MASTL, ARPP19, which shares high sequence homology with ENSA, differentially regulates cell cycle progression and development [17]. These findings highlight the need for a deeper understanding of the specific roles of the MASTL-ENSA pathway during mitosis.

In this study, we elucidated the role of the MASTL-ENSA pathway during mitosis. We provide novel insights into the functional significance of the MASTL-ENSA pathway in maintaining spindle pole integrity through the recruitment of Aurora A during mitosis.

### Material and methods

#### Cell culture and treatment

The HeLa and MCF7 cell lines were obtained from the American Type Culture Collection (ATCC; USA) and the Fucci-HeLa cell line were obtained from RIKEN BioResource Research Center. Dulbecco's modified Eagle's medium (DMEM; Corning) supplemented with 10% fetal bovine serum (FBS; Corning) and 1% penicillin/streptomycin (GeneDepot; Barker, TX, USA) was used for cell culture. The cells were incubated at 37 °C with 5% CO_2_ in a humidified atmosphere. For cell cycle synchronization, 4 × 10^5^ cells were seeded in a 60 mm cell culture dish (Corning), and a double-thymidine (Thy-Thy) block was performed as previously described [18]. MKI-2 synthesized by the Korea Research Institute of Chemical Technology (Daejeon, Korea) was treated at the indicated concentrations for each experiment [19].

### RNA interference

siRNAs targeting ENSA were synthesized by Genolution (Seoul, Korea). The sequence for siENSA #1 was 5’-GCCAAGAUGAAGAAUAAGCUU-3’ and for siENSA #2 was 5’-GAAACAAGAAGAAGAGAACUU-3’. PP2A-B55α siRNAs were purchased from Santa Cruz Biotechnology. Hela cells or Fucci-HeLa cells were plated in 96-well plates overnight. The cells were then transfected with siRNAs using G-fectin (Genolution) following the manufacturer's instructions. After 48 h, the cells were harvested or stained with immunofluorescence antibodies for microscopy.

### Western blotting analysis

Cells were harvested and lysed using RIPA lysis and extraction buffer (GeneDepot), 1% phosphatase inhibitor (GeneDepot), and 1% protease inhibitor cocktail (GeneDepot) on ice for 10 min, and debris was removed by centrifugation. Proteins were separated using 8% SDS-PAGE and transferred onto polyvinylidene difluoride membranes (IPVH0010, Immobilon). The membranes were blocked in TBS-T containing 3% BSA for 1 h at room temperature and then incubated with primary antibodies against β-actin (1:5000, 4967S, Cell Signaling Technology, Danvers, MA, USA), cyclin B1 (1:1000, 4138, Cell Signaling Technology), ENSA (1:1000, GTX101493, GeneTex, CA, USA), pY15-Cdk1 (1:1000, 9111, Cell Signaling Technology), anti-PP2A-B55-α (1:1000, sc-81606), or pT288-Aurora A (1:1000, #3079S, Cell Signaling Technology) overnight at 4 °C. The membranes were washed three times, and then incubated with the appropriate HRP-conjugated anti-rabbit (1:2500, GeneDepot) or anti-mouse (1:2500, GeneDepot) secondary antibodies for 30 min at room temperature. The HRP-conjugated secondary antibody was detected using an enhanced chemiluminescence detection system (Amersham Life Science, Piscataway, NJ, USA). The bands were imaged using an Amersham Imager 600 system (GE Healthcare).

### Immunofluorescence staining

HeLa and MCF7 cells were fixed in 4% paraformaldehyde for 10 min and permeabilized in phosphate-buffered saline (PBS) with 0.1% Triton X-100 and 0.01% Tween 20. After permeabilization, the cells were blocked with 3% bovine serum albumin (BSA) in PBS for 1–2 h at room temperature. The cells were then incubated with primary antibodies such as anti-α-tubulin (1:200, GT114, GeneTex), anti-phosphorylated-Aurora A (1:200, #3079S, Cell Signaling Technology), anti-Pericentrin (1:200, ab4449, Abcam), anti-ENSA (1:200, GTX101493, GeneTex) or anti-phosphorylated-ENSA (1:200, #5240S, Cell Signaling Technology) for 45 min at room temperature. The cells were washed and then incubated with Alexa Fluor 488 conjugated or Alexa Fluor 594-conjugated secondary antibodies. Chromosomes were counterstained with Hoechst. The fluorescence intensity was measured and analyzed using an IN Cell Analyzer 2200 Imaging System (Cytiva, MA, USA) with an automated spherical aberration collar (ASAC) 40X lens and a confocal laser-scanning microscope (LSM 880; Zeiss, Germany) with ZEN 3.3 LSM software (Zeiss, Germany). To quantify the intensity of p-Aurora A in ENSA-inhibited cells, we employed the 'circle' and 'contour' tools within the graphics software 'Zen 3.3' for data analysis. First, we measured the overall green intensity of mitotic cells (A) using the circle tool. Next, we determined the localized green intensity of the spindle pole area (B) using the contour tool. The cytosolic intensity was calculated as (A) minus (B).

### Live cell imaging

For live cell imaging of HeLa cells expressing Fucci, the cells were plated on a cell imaging dish with a glass bottom and cultured in DMEM supplemented with 10% fetal bovine serum. The cells were placed into a growth chamber heated to 37 °C with 5% CO_2_ and observed on an IN Cell Analyzer 2200 Imaging System (Cytiva, MA, USA) with a 20 × lens. Images were acquired every 3 min for 5 h with ZEN 3.3 (Carl Zeiss AG). In the context of data analysis, 'entry initiation' refers to the moment fluorescence signal moves from nucleus to the cytoplasm during interphase. 'Entry completion' signifies the moment when the intensity of the green signal decreases, indicating a successful entry into the M phase and exit from the M phase.

### Proximal Ligation Assay (PLA)

HeLa cells were fixed with 4% paraformaldehyde for 10 min. Subsequently, permeabilization was performed using 0.1% Triton X-100 and 0.01% Tween 20 in phosphate-buffered saline (PBS). The cells were then blocked using Duolink Blocking Solution (DUO92004 and DUO92002; Sigma-Aldrich). Mouse anti-Aurora A (1:100, GTX13824, GeneTex) and rabbit anti-p-ENSA (1:100, 5240S, Cell Signaling Technology) were used as primary antibodies and slides were incubated in a humidified chamber for 1 h at 37 °C. After washing in buffer A (catalog no. DUO82049; Sigma-Aldrich), a ligation solution containing ligase at a 1:40 dilution was added, and slides were incubated in a preheated humidity chamber for 30 min at 37 °C. After washing again in buffer A, an amplification solution containing the polymerase was added at a 1:80 dilution and the slides were then incubated in a preheated humidity chamber for 100 min at 37 °C. Duolink In situ Detection Reagent Orange (DUO92007; Sigma-Aldrich) was used for signal detection. Finally, after washing with 1X PBS, the chromosomes were counterstained with Hoechst.

### Tumor implantation and immunofluorescence

For the syngeneic mouse model, a 4T1-luc cell line (purchased from ATCC) was implanted in BALB/c mice (6-week-old-females) obtained from Orient Bio (Gyeonggi-do, Korea). The mice were injected in the mammary fat pad with 2 × 10^5^ cells, and suspended in a mixture of Matrigel (Corning Life Science, 354,234, NY) and saline (1:3 ratio). For mouse anesthesia, Zoletil (Virbac, France), Rompun (Elanco, US), and a saline solution were mixed at a ratio 2:8:90. Each mouse was intraperitoneally injected with 200 µl in the abdomen for anesthesia. After 1 week of injection, when the tumors reached 100 mm^3^ and stable luminescence expression was confirmed, the mice were randomly assigned to treatment groups containing seven animals: the vesicle (control; 5% DMSO + 55% polyethylene glycol 400 (PEG400; Sigma) + 40% DW); MKI-2 8mpk. MKI-2 was dissolved in a vesicle solution and the animals received MKI-2 (8 mg/kg) five times per week by intraperitoneal (i.p.) injection. Tumor volumes were monitored three times per week using calipers. The mice were killed 28 days after the tumor cells were injected, after which the tumors were removed and weighed. The tumor tissues were embedded in an optimal cutting temperature (OCT) block and processed in cryosection to confirm immunofluorescence. Tumor tissues in the OCT medium were cut into 25 μm sections and placed on slides. Tumor sections were fixed in 4% paraformaldehyde for 10 min and rinsed with PBS. After blocking stage 10% FBS in PBS solution, sections were stained using primary antibodies such as anti-phosphorylated-Aurora A (1:1000, 3079, Cell Signaling Technology), anti-phosphorylated-ENSA (1:50, 5240S, Cell Signaling Technology), anti-phospho-histone H3 (1:200, 9701, Cell Signaling Technology) and anti-cleaved caspase 3 (1:200, 9661, Cell Signaling Technology) at 4 °C overnight. Tumor sections were washed with 0.1% Tween 20 PBS and then treated with the Alexa Fluor 488 conjugated secondary antibodies. Chromosomes were counterstained with Hoechst. The fluorescence intensity was measured and analyzed using a confocal laser-scanning microscope (LSM 880; Zeiss, Germany) with ZEN 3.3 LSM software (Zeiss, Germany). The nuclear size/diameter was measured with computer-assisted image analysis (AxioVision software 4). Nuclear sizes were plotted following computer-assisted imaging analysis of 300 nuclei randomly selected from each group. Animal experiments and procedures were carried out in compliance with the ARRIVE guidelines and all protocols involving mice in this study were reviewed and approved by the IACUC at the Korea Institute of Radiological and Medical Science Animal Center (NO. 2023–0016).

### Statistical analysis

A Student's t-test was performed, and the error bars represent the SE of three independent experiments. A *p*-value < 0.01 (two-tailed) was considered statistically significant.

## Results

### Phosphorylated ENSA is essential for mitotic progression and the maintenance of spindle pole integrity during mitosis

**Fig. 1:**
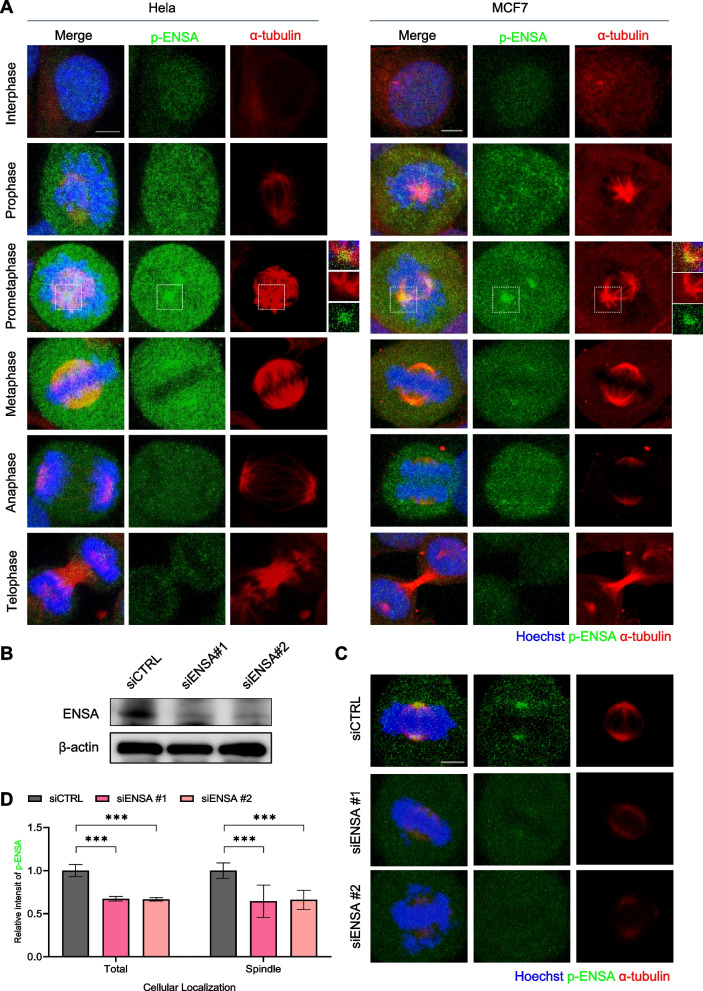
Phosphorylated ENSA localizes at spindle poles during early mitosis. **A** HeLa or MCF cells stained with the indicated antibodies. Spindles and chromosomes were visualized using α-tubulin (red) and Hoechst (blue), respectively. Scale bar, 5 μm. **B** and **C** HeLa cells were transfected with the control or ENSA siRNA (20 nM) for 48 h. Cell lysates were analyzed using immunoblotting with the ENSA antibody (**B**) or stained with the indicated antibodies. **C** Scale bar, 5 μm. **D** Quantification of p-ENSA intensities. The means from three separate experiments are shown. Error bars indicate standard deviation (SD). The data represent typical results and are presented as the mean ± SD of values from three independent experiments; ***, *p* < 0.0001; (*n* = 50 miotic cells for each quantification and group)

To investigate the role of ENSA in mitotic progression, we used immunofluorescence microscopy on HeLa and MCF7 cells to analyze the cellular localization of p-ENSA and ENSA during mitosis. Our results showed that, while ENSA exhibited cytosolic localization (Figure S1), p-ENSA was concentrated at the spindle poles in mitotic cells, implying its potential role at the spindle pole (Fig. 1A). We further validated this finding by transfecting HeLa cells with siRNA against ENSA (siENSA) and observed a significant decrease in the intensity of p-ENSA at the spindle pole in ENSA-depleted prometaphase cells (Fig. 1B–D).

**Fig. 2:**
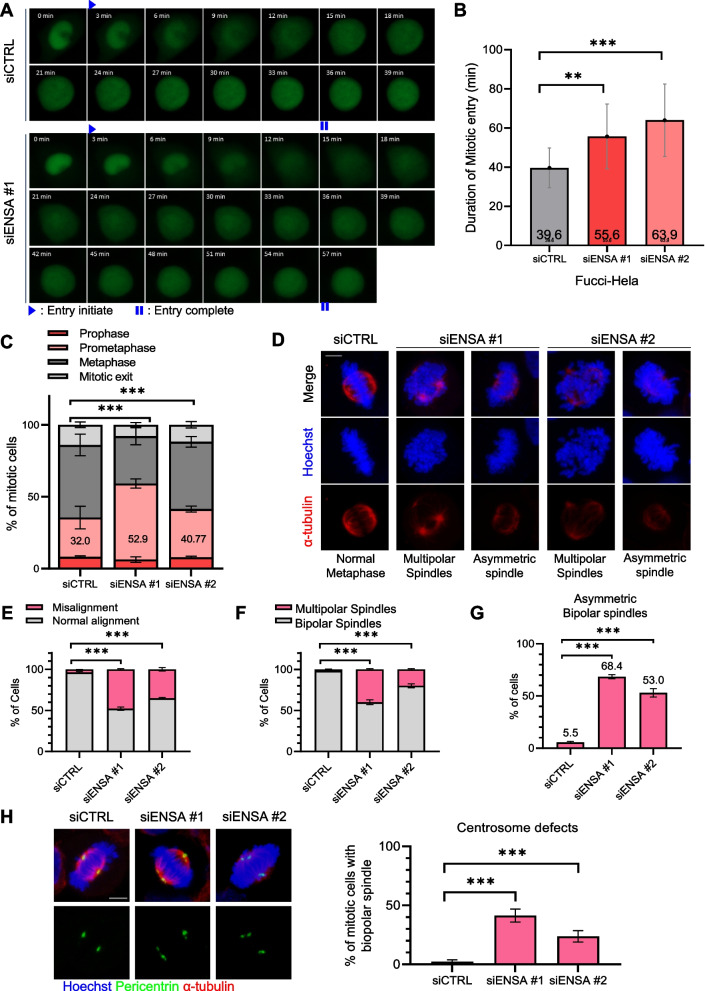
ENSA depletion delays mitotic entry and impairs mitotic spindle pole integrity. **A** Fucci-HeLa cells were transfected with the control or ENSA siRNA. Time-lapse images from 48 h after transfection are shown. The duration from nuclear envelope breakdown (entry initiation) to the formation of the rounded-up shape (entry completion) was determined for the control and ENSA-depleted cells. Images were captured every 3 min to monitor mitotic progression. **B** Quantification of mitotic entry in the control and ENSA-depleted HeLa cells (*n* = 50 cells). Scale bar, 5 μm. Error bar, SEM. **C**-**H** HeLa cells were transfected with the control or ENSA siRNA for 48 h. Quantification of mitotic cells is shown (**C**). Cells were stained with the indicated antibodies (**D**). Scale bar, 5 μm. **E** The percentage of metaphase cells with misaligned chromosomes among the total metaphase cells. **F** Quantification of spindle pole abnormalities during mitosis. **G** Quantification of spindle abnormalities during mitosis. Data are presented as the mean ± SEM of values from at least three independent experiments (*n* = 50 miotic cells for each quantification and group). ***p < 0.0001. **H** Cells were stained with the indicated antibodies (left panel). Scale bar, 5 μm. Quantification of centrosome defects during mitosis (right panel)

To assess the effects of ENSA depletion on mitotic progression, time-lapse experiments were conducted to examine the duration of mitotic entry. We used HeLa cells expressing Fucci, a fluorescence-based cell cycle indicator that enables the visualization of cell cycle progression in living cells [20]. Our results showed that ENSA depletion prolonged the duration between the initiation and completion of mitotic entry and increased the number of prometaphase cells (Fig. 2A–C). Given the enrichment of p-ENSA at the spindle poles, we examined spindle and chromosome morphology. Immunofluorescence analyses revealed that ENSA depletion led to a significant increase in the number of mitotically defective cells, which were characterized by misaligned chromosomes, multipolar spindles, and asymmetric bipolar spindles (Fig. 2D–G). We observed a marked increase in centrosome defects in ENSA-depleted cells with bipolar spindles (Fig. 2H). These findings suggested that ENSA is important for mitotic progression and the maintenance of spindle pole integrity.

### Loss of spindle pole integrity after ENSA inhibition is not associated with PP2A activity

**Fig. 3:**
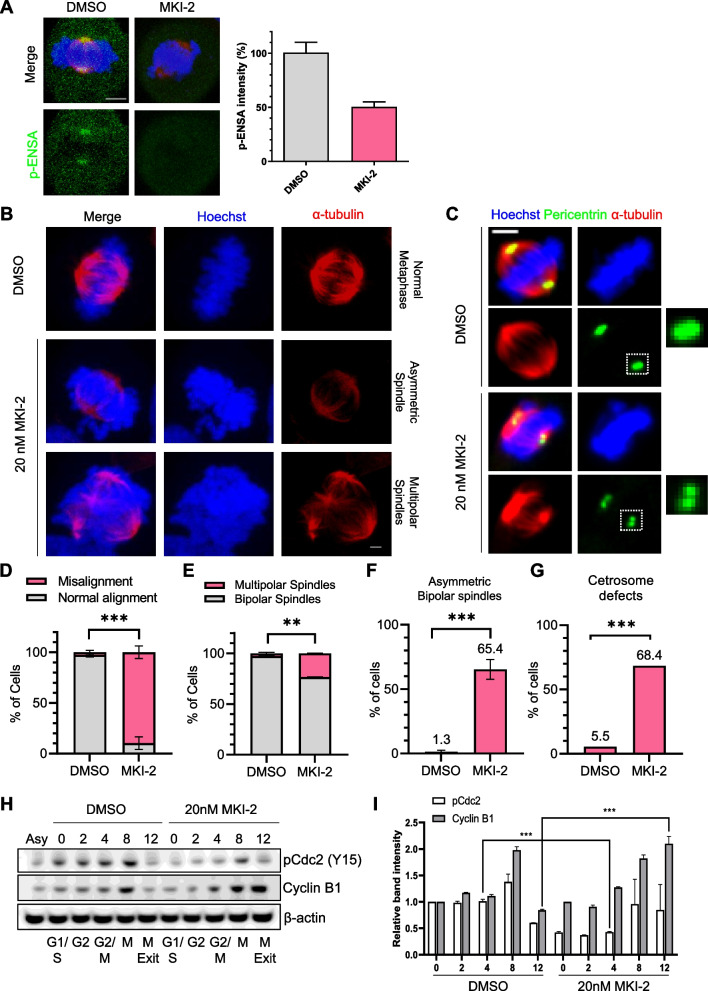
Inhibition of phosphorylated ENSA via MKI-2 delays mitotic entry and disrupts spindle pole integrity. **A**-**C** HeLa cells were incubated in DMSO or 20 nM MKI-2 for 24 h and stained using the indicated antibodies. Spindles and chromosomes were visualized using α-tubulin and Hoechst staining, respectively. Scale bar, 5 μm. **D** The percentage of metaphase cells with misaligned chromosomes among the total metaphase cells. **E** Quantification of spindle pole abnormalities during mitosis. **F** Quantification of spindle abnormalities during mitosis. **G** The percentage of centrosome defects. Data are presented as the mean ± SEM of values from at least three independent experiments (*n* = 50 miotic cells for each quantification and group). ***p < 0.0001; **p < 0.001. **H** and **I** HeLa cells synchronized at the G1/S stage by double thymidine treatment and cultured in DMSO or 20 nM MKI-2 after replacement of the medium. Cells were harvested at the indicated times and analyzed by immunoblotting with the indicated antibodies. Asynchronized cells (Asy) were not arrested by thymidine (**H**). Quantification of the intensities of the indicated protein bands (**I**). The data represent typical results and are presented as the mean ± SD of values from three independent experiments; ***p < 0.0001

To investigate the role of p-ENSA in mitotic progression and spindle pole integrity, we used a specific inhibitor of MASTL kinase, MKI-2 [19]. After synchronization with a double thymidine and thymidine–nocodazole block protocol, HeLa cells were treated with MKI-2, and spindle morphology and mitotic progression were analyzed. MKI-2 significantly decreased p-ENSA levels at the spindle poles, confirming the specificity of the inhibitor and highlighting the importance of MASTL in regulating ENSA phosphorylation (Fig. 3A). MKI-2 treatment increased abnormalities in spindle and chromosome organization and decreased centriole engagement (Fig. 3B–G). MKI-2 treatment delayed the accumulation of p-Cdc2 at 4 h, indicating a G2/M transition delay (Fig. 3H and I). Furthermore, at 12 h, MKI-2 treatment led to an accumulation of Cyclin B1 (Fig. 3H and I), suggesting cell arrest in the mitotic phase due to MKI-2 treatment. These phenotypes were consistent with those observed after ENSA depletion, indicating the specific involvement of the MASTL-ENSA pathway in mitotic progression and spindle pole integrity.

**Fig. 4:**
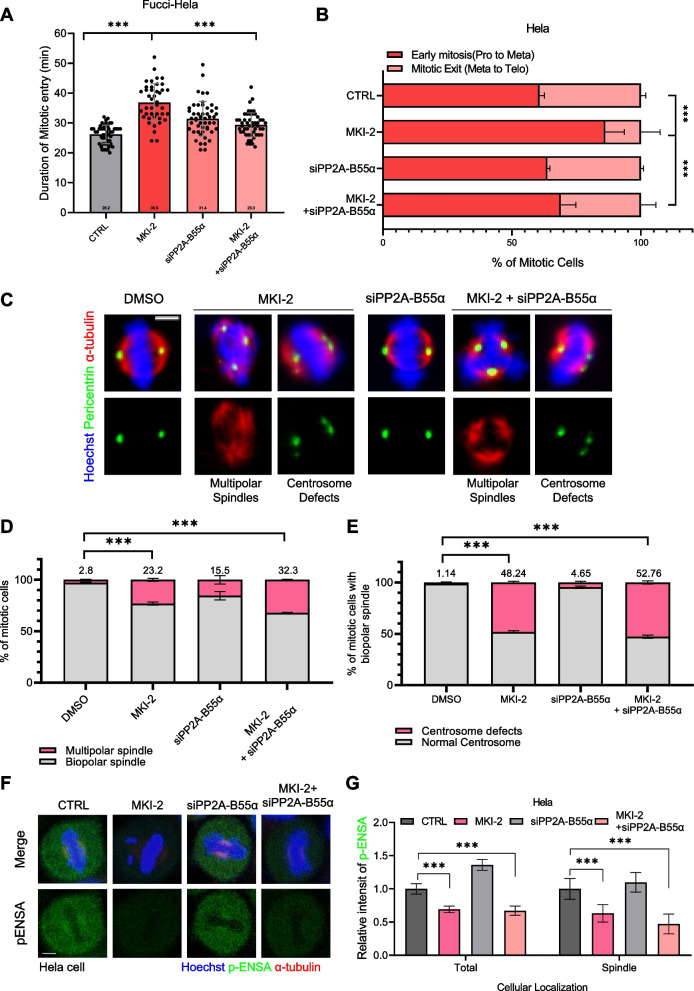
Delay of mitotic entry induced by MKI-2 is restored by PP2A depletion; however, abnormal spindle pole integrity is not affected. **A** The duration of mitotic entry. Fucci-HeLa cells were transfected with the control, PP2A-B55α siRNA in DMSO (Ctrl), or 20 nM MKI-2 and time-lapse imaged from 48 h after transfection and 24 h after MKI-2 treatment. **B**-**G** HeLa cells were treated with DMSO, 20 nM MKI-2, or siRNA of PP2A (5 nM). Quantification of mitotic cells (**B**). HeLa cells were stained with the indicated antibodies (**C** and **F**). Quantification of spindle pole abnormalities during mitosis (**D**). The percentage of centrosome defects (**E**). Data are presented as the mean ± SEM of values from at least three independent experiments (*n* = 50 miotic cells for each quantification and group). ***p < 0.0001. Quantification of p-ENSA intensities (**G**). The means of values from three separate experiments are shown. Error bars indicate standard deviation (SD). The data represent typical results and are presented as the mean ± SD of values from three independent experiments; ***p < 0.0001; (*n* = 100 miotic cells for each quantification and group)

As the MASTL-ENSA pathway negatively regulates PP2A activity during mitotic progression, we investigated whether mitotic delay and spindle pole defects induced by MKI-2 could be reversed by PP2A-B55α depletion [12, 16]. We depleted PP2A-B55α (Figure S2), a key downstream target of MASTL[11, 13, 15, 16, 21]. As expected, our time-lapse analysis of Fucci-labeled HeLa cells showed that MKI-2-induced mitotic delay was restored upon PP2A-B55α depletion (Fig. 4A and B, Figure S2), confirming the involvement of the MASTL-ENSA pathway in mitotic progression in a PP2A-dependent manner. However, the defects in mitotic spindles observed in MKI-2-treated cells, including multipolar spindles and centrosome defects, persisted after PP2A-B55α depletion (Fig. 4C–E). PP2A- B55α depletion alone did not impair spindle pole integrity. This suggested that the MASTL-ENSA pathway has a non-canonical function in regulating spindle pole integrity, operating independently of PP2A- B55α. Notably, PP2A- B55α depletion did not decrease p-ENSA signals at the spindle poles (Fig. 4F and G), confirming the PP2A-independent role of the MASTL-ENSA pathway at the spindle pole.

### p-ENSA interacts with and recruits Aurora A to the spindle poles during mitosis

**Fig. 5:**
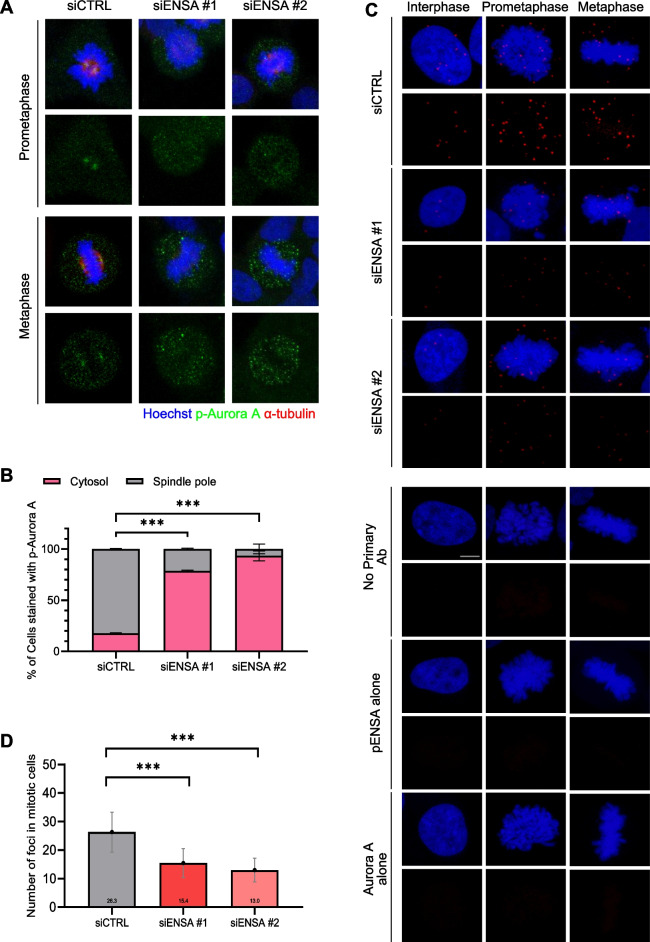
ENSA regulates the recruitment of Aurora A to the spindle pole during mitosis. **A** HeLa cells were transfected with the control or ENSA siRNA for 48 h. Cells were stained with the indicated antibodies. Scale bar, 5 μm. **B** The percentage of cells with p-Aurora A at the cytosol or spindle pole. HeLa cells were transfected with the control or ENSA siRNA for 48 h. (**C** and **D**) PLA of p-ENSA/Aurora A complexes from the control or ENSA-depleted cells. Representative images of the PLA assay (**C**). Number of foci in mitotic cells (**D**). Data are presented as the mean ± SEM of values from at least three independent experiments (*n* = 50 miotic cells for each quantification and group). ***p < 0.0001

Given the critical role of Aurora A in maintaining spindle pole integrity during mitosis [4], we aimed to determine the association between p-ENSA and Aurora A at the spindle poles. We investigated the effect of ENSA inhibition, achieved by siRNA or MKI-2 treatment, on the levels of phosphorylated Aurora A (p-Aurora A) at the spindle poles. Both ENSA inhibition strategies led to a significant reduction in the levels of p-Aurora A at the spindle poles and an increase in the intensity of p-Aurora in the cytosol, although the overall intensity of p-Auroa A during mitosis was not affected (Fig. 5A, B, and Figure S3). These results suggest that the inhibition of ENSA disrupts the recruitment of p-Aurora A at the spindle pole, leading to p-Aurora A remaining in the cytosol. We propose that p-ENSA regulates spindle pole integrity by controlling the maintenance of p-Aurora A at the spindle poles, rather than directly influencing the phosphorylation of Aurora A. For further understanding, we used a proximal ligation assay (PLA) to detect the protein–protein interactions between p-ENSA and Aurora A during interphase and the mitotic phases. While PLA signals remained low in interphase cells, a significant increase was observed in prometaphase and metaphase cells, suggesting interaction between p-ENSA and Aurora A, specifically during mitosis (Fig. 5C, D, and Figure S4). This finding was supported by the decrease in the number of PLA foci in mitotic cells following ENSA depletion, confirming the specificity of the interaction between p-ENSA and Aurora A (Fig. 5C and D). Thus, our results suggested that p-ENSA recruits Aurora A to the spindle pole during mitosis, thereby playing a specific role in maintaining spindle pole integrity.

### MKI-2 induces fatal CIN by suppressing the MASTL-ENSA-Aurora a pathway

**Fig. 6:**
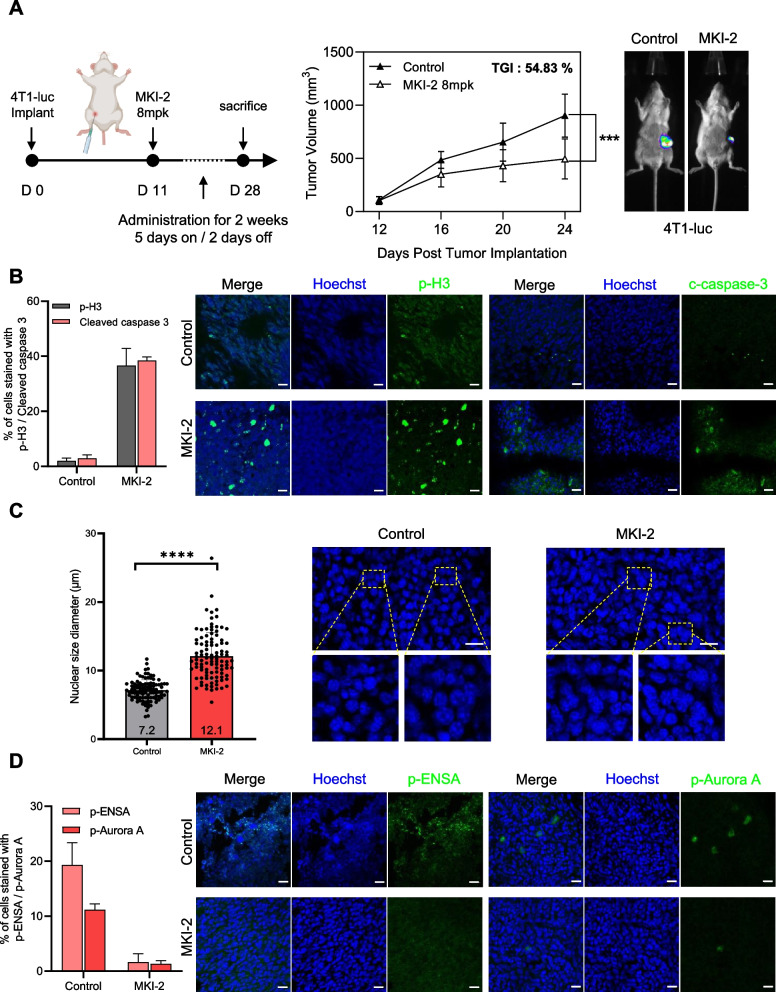
The MASTL inhibitor, MKI-2, sensitizes tumors with increased chromosomal instability and downregulation of MASTL-ENSA-Aurora A. **A** Schematic representation of MKI-2 treatment schedules and tumor volume tracking results. Representative images and quantification of tumor volume of the 4T1-luc syngeneic mouse model treated with vehicle (*n* = 7) or MKI-2 (*n* = 7) for 18 d. **B** Quantification of p-H3 and cleaved caspase 3 intensities (left panel). Tumor tissues were stained with the indicated antibodies (right panel). Scale bar, 20 μm. The means of values from three separate experiments are shown. Error bars indicate standard deviation (SD). The data represent typical results and are presented as the mean ± SD of values from three independent experiments. **C** The nuclear size/diameter was measured using computer-assisted image analysis (AxioVision software 4). Nuclear sizes were plotted following computer-assisted imaging analysis of 300 nuclei randomly selected from each group. Error bars indicate SD. The data represent typical results and are presented as the mean ± SD of values from three independent experiments; ****p < 0.0001. Scale bar, 20 μm. **D** Quantification of p-ENSA and p-Aurora A intensities (left panel). Tumor tissues were stained using the indicated antibodies (right panel). Scale bar, 20 μm. The means of values from three separate experiments are shown. Error bars indicate SD. The data represent typical results and are presented as the mean ± SD of values from three independent experiments

The relationship between spindle pole defects, chromosome missegregation, and the subsequent development of CIN in cancer is well-documented [22]. Moreover, MASTL regulates CIN adaptation to targeted therapy in refractory lethal prostate cancer [23]. Based on our finding that MASTL-ENSA regulates spindle pole integrity by regulating p-Aurora A in cancer cells, we explored the therapeutic potential of targeting the MASTL-ENSA-Aurora A pathway. For this, we used a 4T1 orthotopic breast cancer model. MKI-2 treatment reduced tumor growth in the mouse model (Fig. 6A). MKI-2 induced mitotic cell death, as observed by increased levels of phosphorylated H3, cleaved PARP (Fig. 6B), and CIN (Fig. 6C). MKI-2 decreased the levels of p-ENSA and p-Aurora A in the breast cancer model (Fig. 6D). Therefore, our data suggested that MASTL regulates CIN adaptation by modulating mitosis via the MASTL-ENSA-PP2A-B55α pathway and by regulating spindle pole integrity via the MASTL-ENSA-Aurora A pathway.

## Discussion

At the onset of mitosis, the accumulation of cyclin B triggers the activation of CDK1, which consequently phosphorylates various proteins involved in mitosis [14]. MASTL, a key regulator of mitotic progression, phosphorylates ENSA and ARPP19, which subsequently inhibits the activity of PP2A-B55, a protein phosphatase that counteracts the effects of cyclin B-CDK1 [10, 11]. Therefore, inhibition of PP2A-B55 is essential for sustaining the activity of cyclin B-CDK1 during mitosis. Although the MASTL-ENSA pathways are well-established regulators of PP2A-B55 activity during mitotic progression [10, 11, 13, 24], other targets of MASTL-ENSA remain unidentified. Here, we provide the first evidence that p-ENSA is concentrated at the spindle poles during mitosis and plays a crucial role in controlling spindle pole integrity independent of PP2A. Notably, we found that p-ENSA interacts with Aurora A, thereby helping maintain Aurora A to the spindle poles. These findings revealed a non-canonical function of the MASTL-ENSA pathway in maintaining spindle pole integrity during mitosis.

**Fig. 7:**
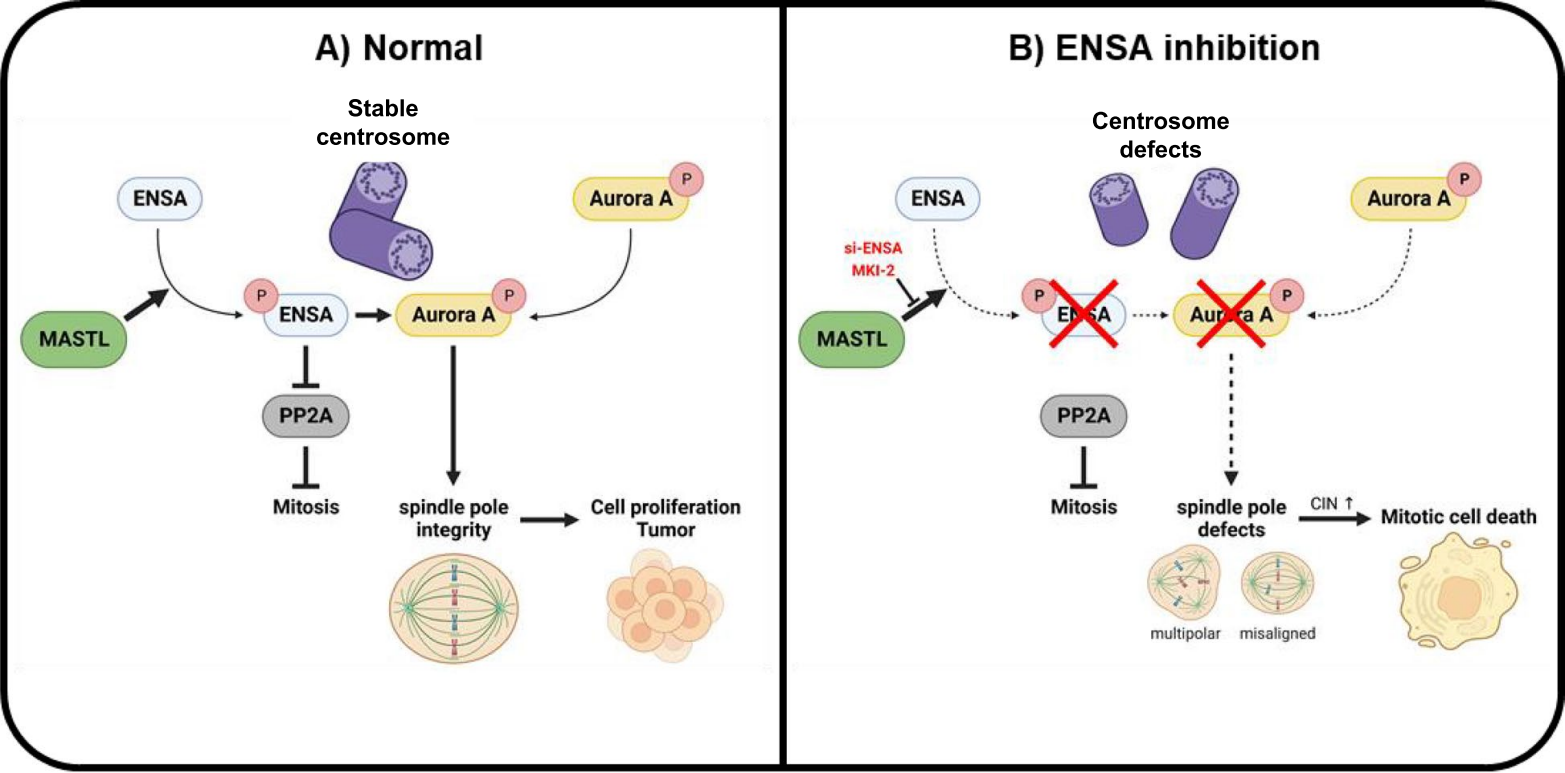
Graphic summary of the regulation of the MASTL-ENSA-Aurora A pathway during mitosis

The role of the MASTL-ENSA-PP2A-B55 axis in mitotic entry and exit has been well documented [10, 11, 13, 24]. However, our data revealed a novel aspect of the MASTL-ENSA pathway by revealing its involvement in spindle pole integrity via its interaction with Aurora A. ENSA depletion induces severe mitotic defects, including increased centrosome defects and mitotic delay. These findings indicated that ENSA regulates mitotic progression and spindle pole integrity in human cells. Regarding the role of ENSA in spindle pole integrity, MASTL-knockout mouse embryonic fibroblasts reduce the phosphorylation of MPS1, a key spindle assembly checkpoint (SAC) kinase, suggesting that MASTL inhibition impairs the SAC [25]. Furthermore, MASTL overexpression is associated with CIN in breast and metastatic prostate cancers [23, 26]. While it is possible that the impaired SAC and CIN observed after MASTL knockout and overexpression, respectively, were associated with dysregulated PP2A activity, these defects could be mediated by the MASTL-ENSA-Aurora A pathway. This is supported by the fact that Aurora A regulates SAC activity and has implications for oncogenic transformation during CIN development [27, 28]. Notably, the phosphorylation of NuMA, a key protein involved in the organization of mitotic spindle poles and control of spindle orientation, is modulated by PP2A-B55, independent of the MASTL-ENSA pathway [29]. This aligned with our finding that the role of ENSA in spindle pole integrity is independent of PP2A-B55α activity. Considering the role of Aurora A in spindle assembly and chromosome segregation [4], we proposed that the diverse mitotic events associated with the MASTL-ENSA pathway are regulated by both Aurora A and PP2A-B55 (Fig. 7). In addition, future studies are required to understand the regulatory interplay between ENSA and various mitotic regulators, which will further enhance our understanding of MASTL-ENSA's role in spindle pole integrity.

MASTL is a promising anticancer target, particularly in cancers with CIN, such as triple-negative breast cancer and metastatic prostate cancer [13, 23, 26]. There are several pharmacological inhibitors of MASTL, including GKI-1, MKI-1, and MKI-2 [15, 19, 30]. Among them, we demonstrated that MKI-2, the most potent MASTL inhibitor [19], markedly increases mitotic cell death and induces spindle defects in HeLa and MCF7 cells, and in a 4T1 breast tumor model. These findings indicate that the pharmacological inhibition of MASTL inhibits mitotic progression and induces spindle instability, leading to reduced cancer growth. Notably, MASTL inhibits fatal levels of CIN and promotes CIN tolerance in lethal therapy-resistant prostate cancer [23]. In addition, in silico modeling has shown that MASTL stabilizes Aurora A in neuroblastoma cells [31]. Similarly, our data suggest that pharmacological MASTL inhibitors increase spindle pole defects by inhibiting the MASTL-ENSA-Aurora A regulatory pathway. This, consequently, could induce deleterious CIN in CIN-resistant cancers, highlighting the potential of pharmacological MASTL inhibitors as therapeutic agents that induce fetal CIN in cancer.

## Conclusion

In summary, this study identified a non-canonical function of MASTL-ENSA in preserving spindle pole integrity, highlighting its significance in mitosis. Our findings provide new insight into the regulation of spindle pole integrity during mitosis via the MASTL-ENSA-Aurora A pathway.

### Supplementary Information

**Additional file 1: Figure S1. **ENSA localizes at the cytosol and not at the spindle poles during mitosis. (A-B) HeLa and MCF cells were stained using the indicated antibodies. Spindles and chromosomes were visualized using α-tubulin (red)(A) or γ-tubulin (red)(B) and Hoechst (blue), respectively. Scale bar, 5 μm. **Figure S2. **MKI-2-induced mitotic delay is restored upon PP2Aα depletion. (A) Fucci-HeLa cells were transfected with the control, PP2A-B55α siRNA in DMSO (Ctrl), or 20 nM MKI-2. A time-lapse image from 48 h after transfection is shown. The duration between nuclear envelope breakdown (entry initiation) and the formation of the rounded-up shape (entry completion) was determined. Images were captured every 3 min to monitor mitotic progression. (B) MCF7 cells were transfected with the control or PP2A-B55α siRNA (5nM) in DMSO. Protein level of PP2A-B55α was analyzed by immunoblotting with the indicated antibodies. **Figure S3. **ENSA regulates the recruitment of Aurora A to the spindle pole during mitosis. Percentage of cells with p-Aurora A in the cytosol or spindle pole. HeLa cells were treated with DMSO or 20 nM MKI-2 for 24 h. The cells were stained using the indicated antibodies. Scale bar, 5 μm.** Figure S4. **ENSA interacts with Aurora A during mitosis. PLA of p-ENSA/Aurora A complexes from the control or ENSA-depleted cells. Number of foci in interphase cells. Data are represented as the mean ± SEM of values from at least three independent experiments (*n* ≥ 50 interphase cells for each quantification and group) ****p < 0.0001. **Figure S5. **Uncropped western blot membranes.

## Data Availability

All data generated or analyzed during this study are included in this published article.
