# Supplementary material for: Endosulfine alpha maintains spindle pole integrity by recruiting Aurora A during mitosis
Source: BMC Cancer. 2023 Dec 21;23:1263. doi: 10.1186/s12885-023-11742-0 (PMC10734108; doi:10.1186/s12885-023-11742-0)
Supplement: Supplementary file 1 — Additional file 1: Figure S1. ENSA localizes at the cytosol and not at the spindle poles during mitosis. (A-B) HeLa and MCF cells were stained using the indicated antibodies. Spindles and chromosomes were visualized using α-tubulin (red)(A) or γ-tubulin (red)(B) and Hoechst (blue), respectively. Scale bar, 5 μm. Figure S2. MKI-2-induced mitotic delay is restored upon PP2Aα depletion. (A) Fucci-HeLa cells were transfected with the control, PP2A-B55α siRNA in DMSO (Ctrl), or 20 nM MKI-2. A time-lapse image from 48 h after transfection is shown. The duration between nuclear envelope breakdown (entry initiation) and the formation of the rounded-up shape (entry completion) was determined. Images were captured every 3 min to monitor mitotic progression. (B) MCF7 cells were transfected with the control or PP2A-B55α siRNA (5nM) in DMSO. Protein level of PP2A-B55α was analyzed by immunoblotting with the indicated antibodies. Figure S3. ENSA regulates the recruitment of Aurora A to the spindle pole during mitosis. Percentage of cells with p-Aurora A in the cytosol or spindle pole. HeLa cells were treated with DMSO or 20 nM MKI-2 for 24 h. The cells were stained using the indicated antibodies. Scale bar, 5 μm. Figure S4. ENSA interacts with Aurora A during mitosis. PLA of p-ENSA/Aurora A complexes from the control or ENSA-depleted cells. Number of foci in interphase cells. Data are represented as the mean ± SEM of values from at least three independent experiments (n ≥ 50 interphase cells for each quantification and group) ****p < 0.0001. Figure S5. Uncropped western blot membranes. [file 12885_2023_11742_MOESM1_ESM.docx]

**
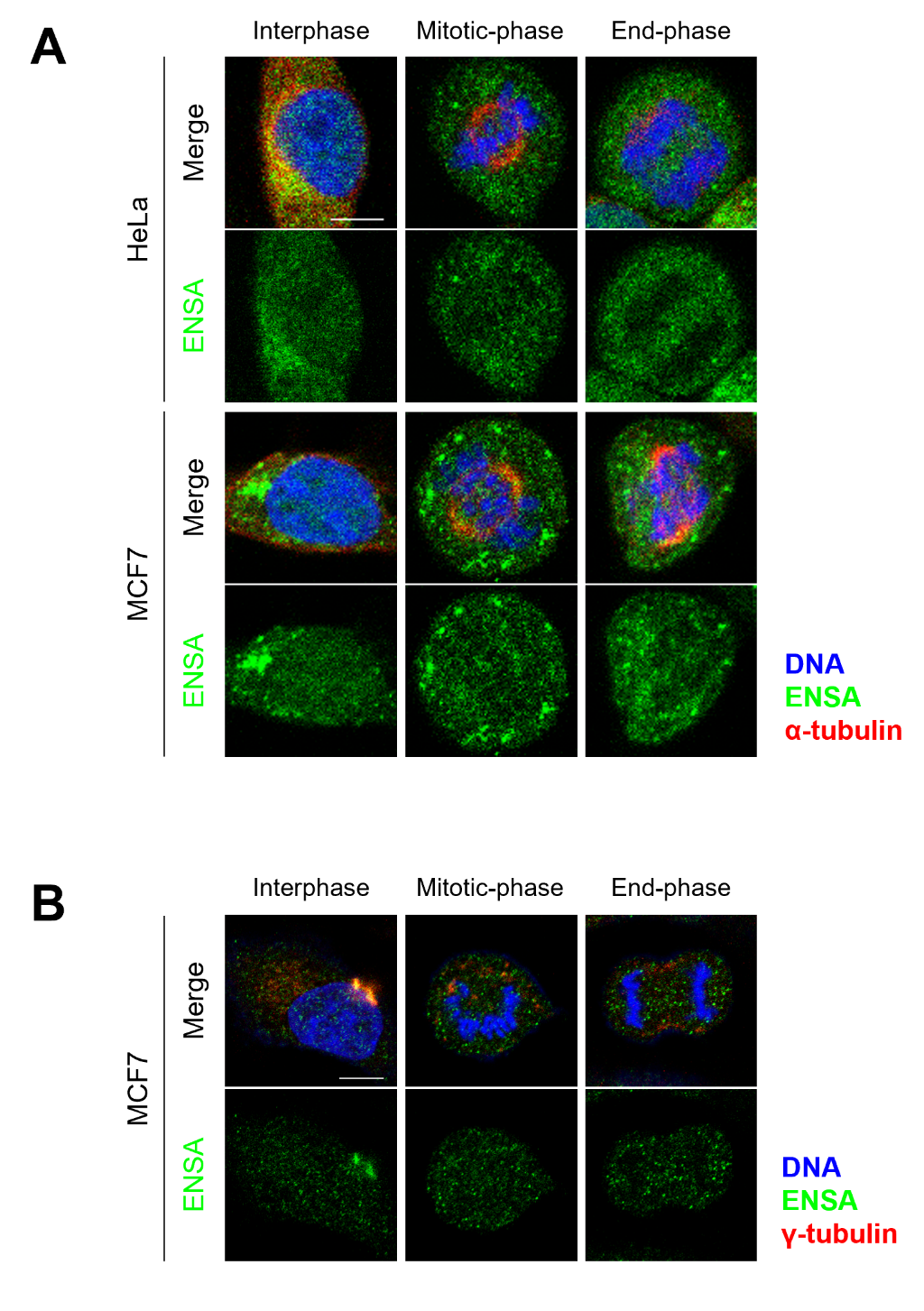
**

**Figure S1. ENSA localizes at the cytosol and not at the spindle poles during mitosis.**

(A-B) HeLa and MCF cells were stained using the indicated antibodies. Spindles and chromosomes were visualized using α-tubulin (red)(A) or γ-tubulin (red)(B) and Hoechst (blue), respectively. Scale bar, 5 μm.

**
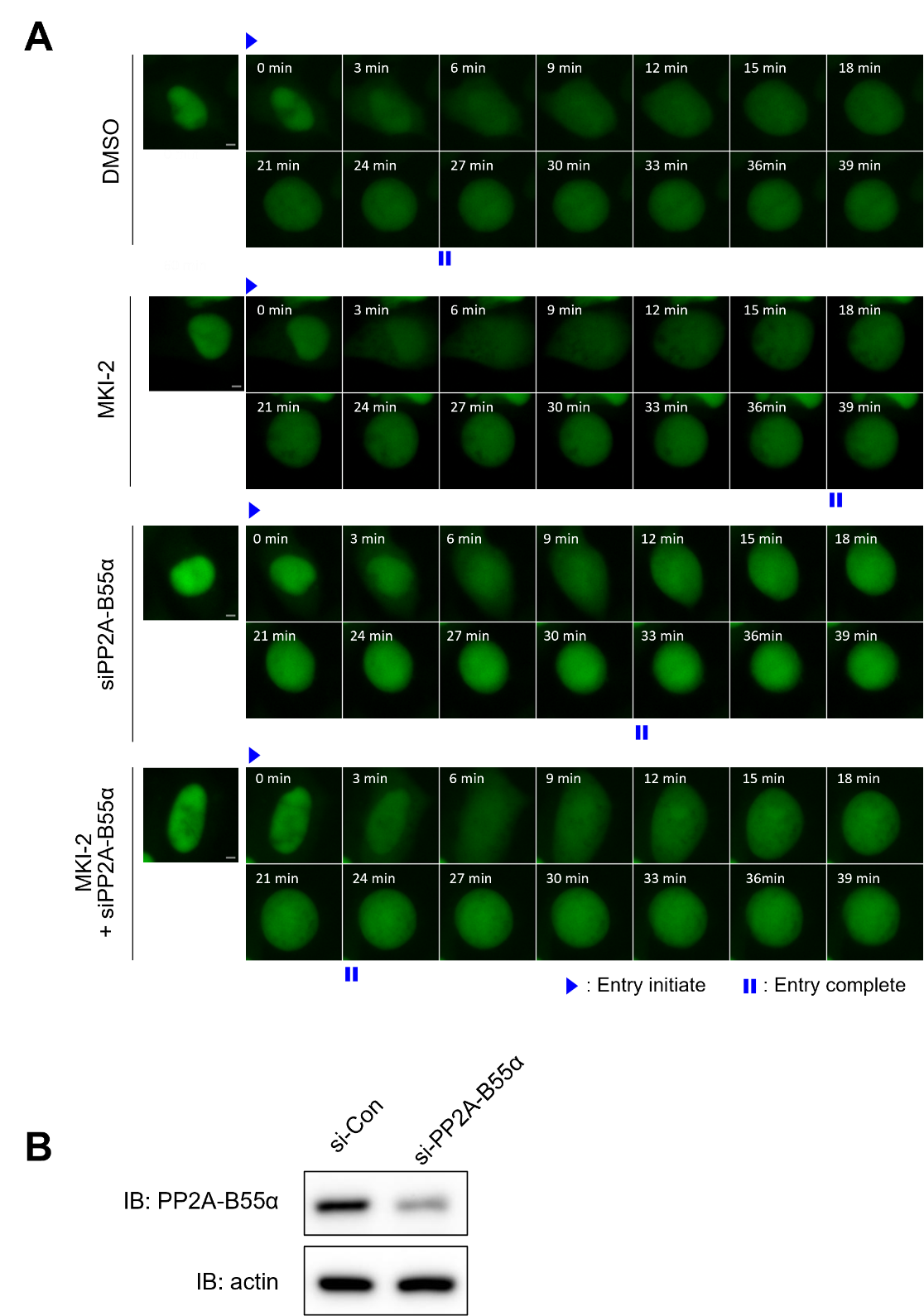
**

**Figure S2. MKI-2-induced mitotic delay is restored upon PP2Aα depletion.**

(A) Fucci-HeLa cells were transfected with the control, PP2A-B55α siRNA in DMSO (Ctrl), or 20 nM MKI-2. A time-lapse image from 48 h after transfection is shown. The duration between nuclear envelope breakdown (entry initiation) and the formation of the rounded-up shape (entry completion) was determined. Images were captured every 3 min to monitor mitotic progression.

(B) MCF7 cells were transfected with the control or PP2A-B55α siRNA (5nM) in DMSO. Protein level of PP2A-B55α was analyzed by immunoblotting with the indicated antibodies.


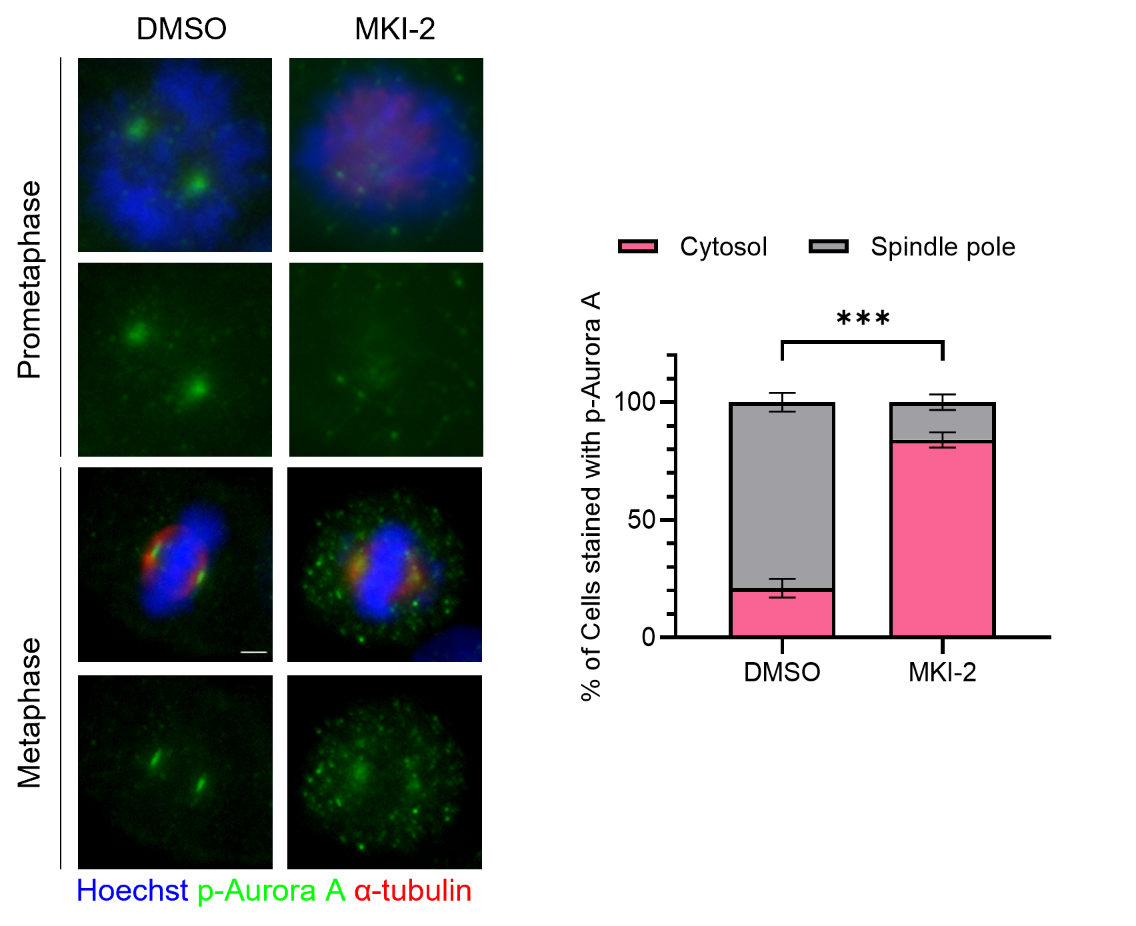


**Figure S3. ENSA regulates the recruitment of Aurora A to the spindle pole during mitosis.**

Percentage of cells with p-Aurora A in the cytosol or spindle pole. HeLa cells were treated with DMSO or 20 nM MKI-2 for 24 h. The cells were stained using the indicated antibodies. Scale bar, 5 μm.


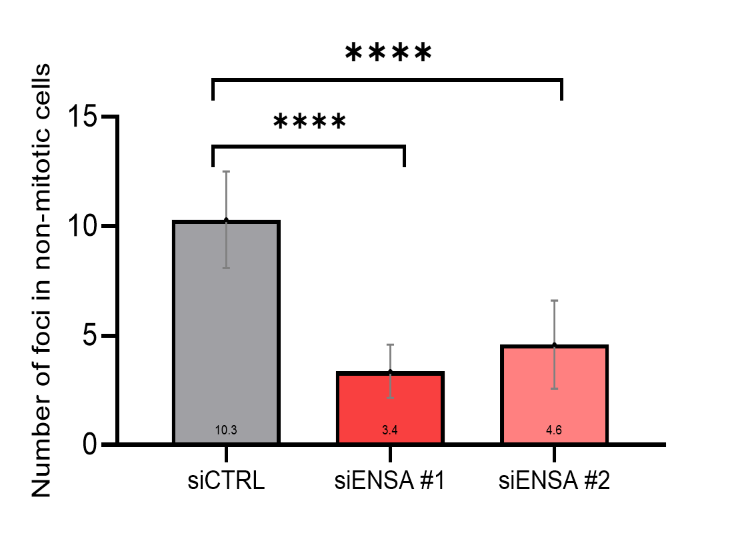


**Figure S4. ENSA interacts with Aurora A during mitosis.**

PLA of p-ENSA/Aurora A complexes from the control or ENSA-depleted cells. Number of foci in interphase cells. Data are represented as the mean ± SEM of values from at least three independent experiments (n ≥ 50 interphase cells for each quantification and group) ****p < 0.0001.


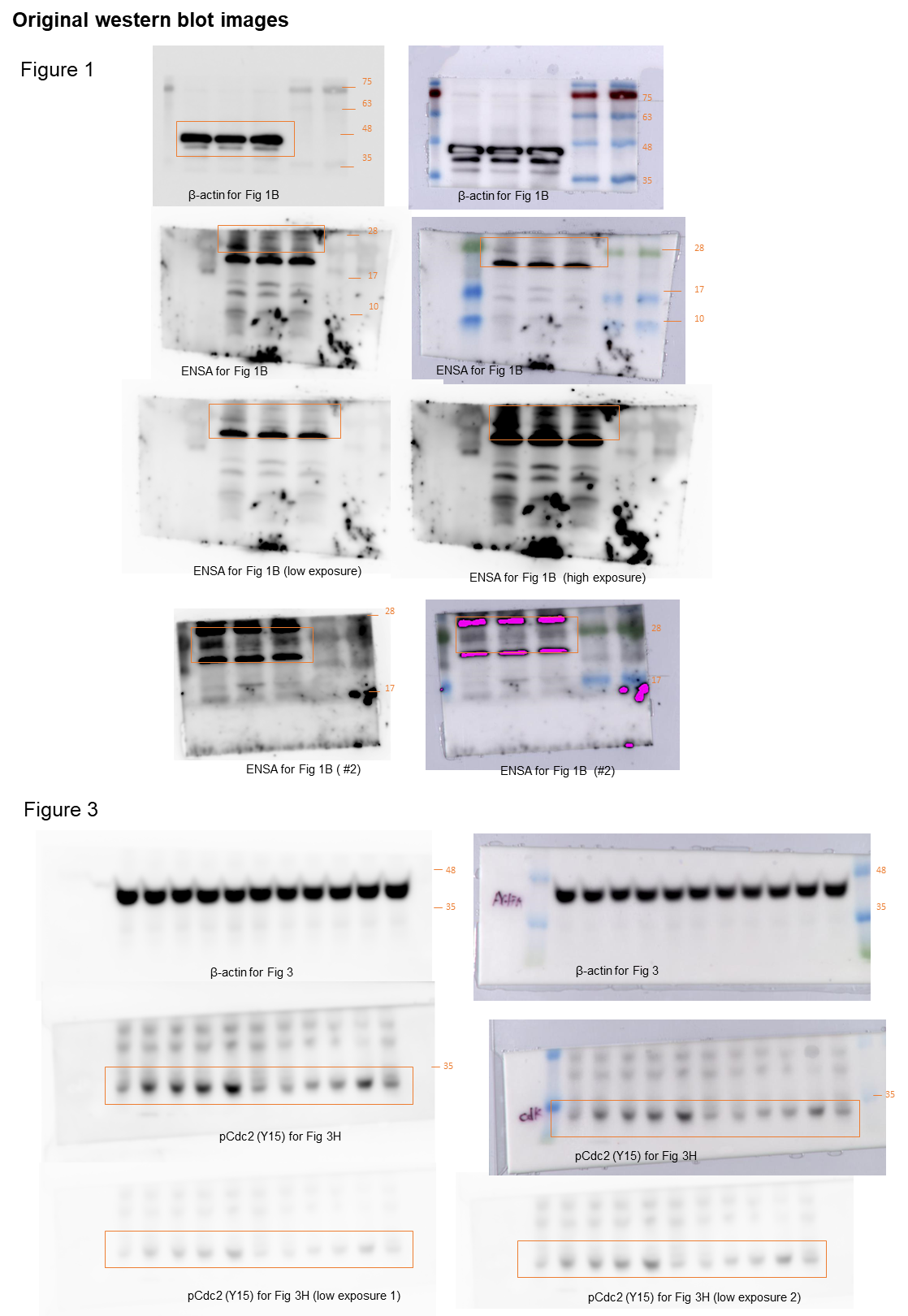


**
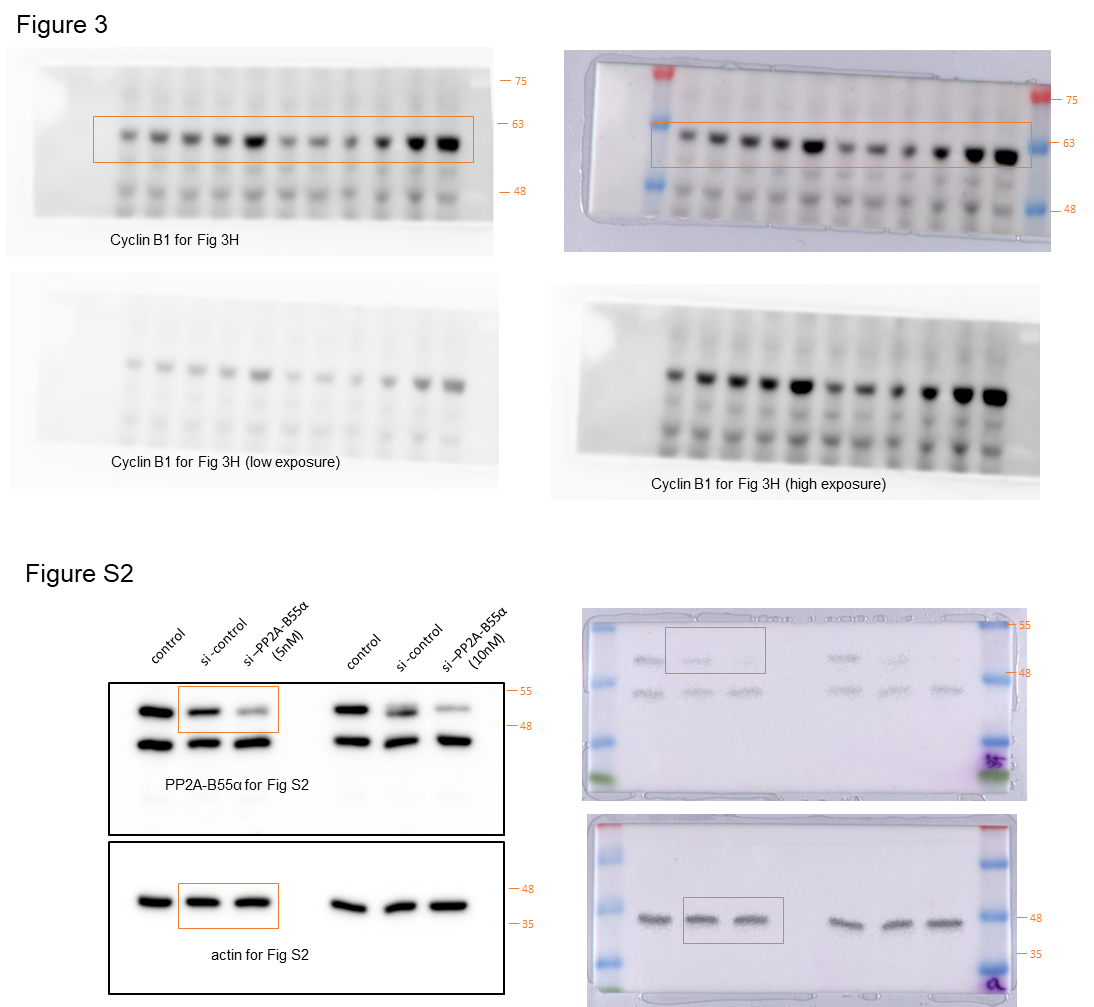
Figure S5.** Uncropped western blot membranes.
